# Examining Predictors of Depression and Anxiety Symptom Change in Cognitive Behavioral Immersion: Observational Study

**DOI:** 10.2196/42377

**Published:** 2023-07-14

**Authors:** Iony D Ezawa, Steven D Hollon, Noah Robinson

**Affiliations:** 1 Department of Psychology University of Southern California Los Angeles, CA United States; 2 Department of Psychology Vanderbilt University Nashville, TN United States

**Keywords:** Cognitive Behavioral Immersion, virtual reality, metaverse, alliance, social support, cognitive behavioral, depression, anxiety, mood, mental health, mobile phone

## Abstract

**Background:**

Depressive and anxiety disorders are the most common mental disorders, and there is a critical need for effective, affordable, and accessible interventions. Cognitive Behavioral Immersion (CBI) is a novel group-based cognitive behavioral skills training program delivered by lay coaches in the metaverse that can be accessed through various modalities including virtual reality (VR) head-mounted displays or flat-screen devices. Combining its ability to offer empirically supported therapy skills in a digital setting that can still facilitate interpersonal variables (eg, working alliance and sense of social support) with the aid of lay coaches, CBI has the potential to help fill this critical need.

**Objective:**

This study had 2 primary aims. First, we aimed to examine changes in depression and anxiety symptoms in a sample of individuals who participated in CBI. Second, we aimed to examine 2 interpersonal process variables (working alliance and web-based social support) as predictors of symptom changes. We predicted CBI participants would experience depression and anxiety symptom improvements and that such improvements would be associated with an increase in both interpersonal process variables.

**Methods:**

The study sample consists of 127 participants who endorsed clinical levels of depression or anxiety symptoms during their first CBI session and attended at least 2 sessions. Participants were asked to complete self-report measures of depression symptoms, anxiety symptoms, alliance, and web-based social support throughout their participation in CBI.

**Results:**

Repeated measures ANOVAs determined that depression and anxiety symptom scores differed significantly across sessions (*P*s<.01). We also found participants’ web-based social support predicted improvement in depression symptoms (*P*=.01), but neither the alliance nor web-based social support predicted change in anxiety symptoms (*P*s>.05). We also observed a significant difference in anxiety symptoms between participants who used a VR head-mounted display to access CBI and those who did not, such that participants who used VR head-mounted displays endorsed lower anxiety symptoms than those who did not at nearly every session (*P*=.04).

**Conclusions:**

Participation in CBI is associated with both depression and anxiety symptom improvement. Web-based social support may play an important role in fostering changes in depression symptoms. Future studies are encouraged to continue examining the process of change in CBI with special attention paid to methods that can elucidate causal mechanisms of change.

## Introduction

Depressive and anxiety disorders are the most common mental disorders, with an annual estimated 280 million people living with a depressive disorder and 301 million people living with an anxiety disorder [1]. Symptoms of depression and anxiety contribute to detriments in well-being, health, and functioning, and rank among the leading public health burdens worldwide [2]. While there are effective treatments for each, only a portion of those with these conditions receive treatment. There is a critical need for effective, affordable, and accessible interventions.

Cognitive behavioral therapy (CBT) is one of the most effective tools for the treatment of depression and anxiety. CBT is based on a model of how one’s thoughts, emotions, behaviors, and physiology interact in one’s environment [3]. According to the model, a person can indirectly change their emotions by changing maladaptive cognitions, behaviors, or physiological states. In this approach, therapists teach clients a host of cognitive and behavioral tools to identify, evaluate, and correct inaccurate negative thoughts, and increase behaviors that build efficacy and agency and boost positive affect [4]. In the treatment of depression, CBT has been rigorously tested in clinical trials and has been shown to be as efficacious as medications [5] and to have an enduring effect that lasts beyond the end of treatment [6]. CBT has also been shown to be efficacious and have enduring effects in the treatment of anxiety disorders [7]. Despite their efficacy, CBT-oriented interventions are often very expensive and difficult to access. Further, because of the stigma associated with psychological conditions, many individuals do not present for help [8].

To address problems of affordability and accessibility, the field is increasingly using lay therapists (also commonly referred to as coaches or peers) to provide cognitive behavioral coaching. Investigations of lay therapists provide empirical support that well-trained coaches can be trained to deliver effective cognitive behavioral interventions [9-11]. The use of lay therapists offers a way to reach millions of affected individuals who might otherwise not receive any care.

There also is increasing evidence suggesting that technology can be used to deliver affordable and accessible treatments with outcomes comparable to traditional face-to-face psychotherapy interventions [12]. In line with this, digital treatments are increasingly being developed and used as the “new normal” in a pandemic and postpandemic world. While some digital CBT programs have come in the form of self-guided computerized programs that waive the need for therapists altogether, a recent meta-analysis reported that the efficacy and engagement in digital CBT programs are enhanced when provided with coach- or peer-support via email or phone communication [12].

Opportunely, internet-connected virtual environments have now been developed that allow participants to interact with one another as avatars. These virtual environments are often referred to as the “metaverse” [13]. Most of these environments are videogames (eg, Fortnite, Minecraft, Roblox, SecondLife, and World of Warcraft) accessed through flat screens such as mobile phones or desktop computers. More recently, the proliferation of consumer virtual reality (VR) head-mounted displays extends experiences in the metaverse by further immersing individuals into virtual environments in which they can think, feel, and behave as if the virtual environment they are in is real [14]. Moreover, the use of VR to connect to the metaverse can simultaneously promote a sense of presence (ie, the subjective experience of being in a place or environment when one is physically situated in another) [15] and maintain a sense of anonymity via use of an avatar. In other words, participants can still reap the benefits of feeling as if they are in a shared social space with others while also benefitting from a veil of anonymity, which can keep them safe from the perceived judgment from others that may otherwise prevent them from trying out new skills, meeting new people, and taking therapeutic risks such as disclosing their own vulnerabilities.

Although there is a robust literature that demonstrates evidence for the use of VR without connecting to the metaverse in mental health treatment (specifically in treating posttraumatic stress disorder and specific phobias) [16,17], there remains a gap in the literature exploring the use of VR metaverse interventions for any mental health concern. Immersion into the metaverse may be an especially powerful modality to facilitate mental health programs. Besides acting as a digital medium for providing empirically supported cognitive behavioral skills training, the metaverse may help facilitate the nonspecific effects that help account for symptom change in traditional in-person psychological treatments [18]. Specifically, the interpersonal nature of the metaverse may help foster a working alliance between coaches and participants as well as nurture a broader sense of web-based social support. Each of these interpersonal elements has been shown to play a key role in promoting therapeutic outcomes in traditional in-person interventions [19,20], and the metaverse may be a uniquely positioned setting in which each of these components can be introduced digitally to help reduce depression and anxiety symptoms.

Cognitive behavioral immersion (CBI; accessed via the Innerworld app developed by Innerworld, Inc) is a novel synchronous cognitive behavioral skills training group delivered by coaches in the metaverse [13]. CBI was developed to combine the strengths of recent psychological and technological advances to potentially deliver efficacious, affordable, accessible, and scalable mental health support. Upon entering the Innerworld app, participants create an anonymous username and embody a custom, self-designed avatar to attend live events that are hosted by coaches trained in CBI. Participants communicate with one another by speaking out loud, and the audio is transmitted over the internet in real time. Besides voice communication, other social cues are communicated through head movement, hand movement, and emojis (used to communicate affect). CBI is accessible through various modalities, including VR technology (ie, Oculus Quest head-mounted displays) and non-VR modalities (ie, mobile phones and desktop computers).

In this study, we had 2 primary aims. First, we aimed to examine changes in depression and anxiety symptoms among a group of individuals who participated in CBI. Given the novelty of the interpersonal aspects of the metaverse, our second aim was focused on examining 2 interpersonal variables (working alliance and web-based social support) as predictors of symptom change. In line with our first aim, we predicted participants would experience depression and anxiety symptom improvement throughout their participation in CBI. In line with our second aim, we predicted higher ratings of working alliance and perceived sense of web-based social support would be related to greater symptom improvement. In addition to these primary aims, we explored whether the magnitude of symptom change differed based on the type of device (VR vs non-VR) participants used.

## Methods

### Participants

**Figure 1:**
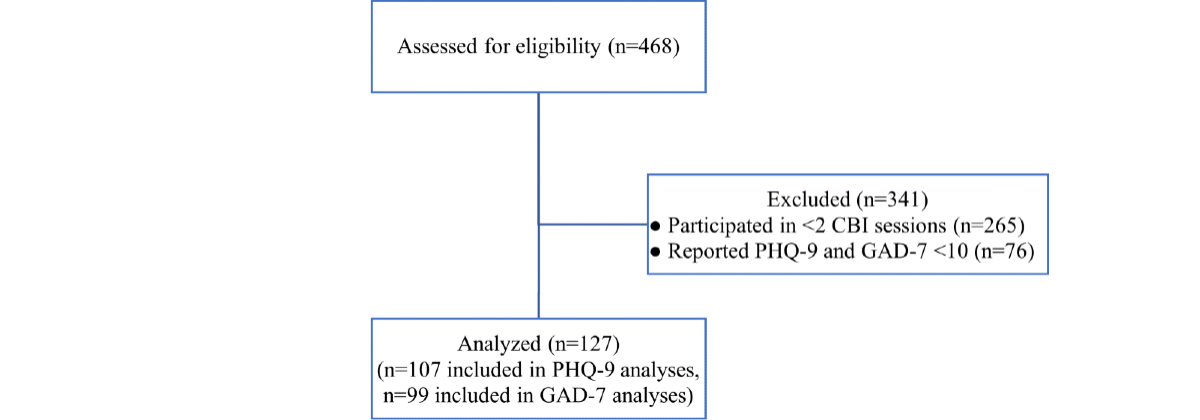
Flowchart of study participant recruitment. CBI: Cognitive Behavioral Immersion; GAD-7: 7-item Generalized Anxiety Disorder scale; PHQ-9: 9-item Patient Health Questionnaire.

Participants were selected based on the following criteria: (1) participated in at least 2 CBI sessions, (2) completed the symptom measures, and (3) reported a clinical level of depression or anxiety symptoms at their first CBI session (as evidenced by scoring a 10 or greater on the 9-item Patient Health Questionnaire (PHQ-9) [21] or 7-item Generalized Anxiety Disorder scale (GAD-7) [22]. A total of 127 participants met the study criteria out of the larger number of 468 who participated in at least 1 session of CBI. Specifically, 107 participants met the study criteria while reporting a clinical level of depressive symptoms, whereas 99 participants met study criteria while reporting a clinical level of anxiety symptoms (including 79 participants who reported clinical levels of both). See Figure 1 for a flowchart of participant recruitment.

Collectively, these participants were predominantly White, single, and employed males aged between 30 and 39 years. Participants attended an average of 13.80 (SD 26.61; median 6; mode 2) sessions, and approximately 79% (100/127) of the sample accessed CBI using a VR head-mounted display. See Table 1 for additional information on participant characteristics.

**Table 1:** Participant characteristics.

Variable		Participants, n (%)
**Age (years)**		
	18-20	16 (12.6)
	21-29	29 (22.8)
	30-39	35 (27.6)
	40-49	27 (21.3)
	50-59	11 (8.7)
	60-69	3 (2.4)
	No response	6 (4.7)
**Gender**		
	Female	43 (33.9)
	Male	68 (53.5)
	Other	9 (7.1)
	No response	7 (5.5)
**Race**		
	American Indian or Alaskan	2 (1.6)
	Asian	13 (10.2)
	Black or African American	5 (3.9)
	Other	10 (7.9)
	White	89 (70.1)
	No response	8 (6.3)
**Marital status**		
	Divorced	10 (7.9)
	Married	31 (24.4)
	Separated	3 (2.4)
	Single	75 (59.1)
	Widowed	1 (0.8)
	No response	7 (5.5)
**Employment status**		
	Disabled, not able to work	9 (7.1)
	Full-time employed	53 (41.7)
	Part-time employed	21 (16.5)
	Not employed, searching for work	11 (8.7)
	Not employed, not searching for work	23 (18.1)
	Retired	2 (1.6)
	No response	8 (6.3)
**Income (US $)**		
	20,000	38 (29.9)
	20,000-49,999	39 (30.7)
	50,000-74,999	20 (15.8)
	≥75,000	17 (13.4)
	No response	13 (10.2)
**Main device used to access CBI^a^**		
	VR head-mounted display	100 (78.7)
	Non-VR device (phone or computer)	27 (21.3)
**Sessions attended**		
	2	23 (18.1)
	3	15 (11.8)
	4	14 (11.0)
	5	11 (8.7)
	6	4 (3.2)
	7	6 (4.7)
	8	6 (4.7)
	9	6 (4.7)
	10	5 (3.9)
	≥11	37 (29.1)

^a^CBI: Cognitive Behavioral Immersion.

### Measures

#### Demographics Survey

Participants were asked to complete a self-report survey assessing a variety of demographic variables including age, gender, race, marital status, employment status, and annual household income. This survey was administered upon entering the Innerworld app for the first time.

#### PHQ-9

The PHQ-9 [21] is a 9-item self-report measure based on the diagnostic criteria for major depressive disorder from the Diagnostic and Statistical Manual Fourth Edition. Each item is scored on a 0- to 3-point basis, and total scores can range from 0 to 27 with greater scores indicating greater severity of depression symptoms. A cutoff score of at least 10 has been recommended to detect clinical levels of depression symptom severity [23]. Participants were asked to complete this measure after each CBI session. Internal consistency for PHQ-9 scores in this study was acceptable (α=.72 at the first session).

#### GAD-7

The GAD-7 [22] is a 7-item self-report measure based on the diagnostic criteria for generalized anxiety disorder from the Diagnostic and Statistical Manual Fourth Edition. It has been used in primary care and research settings to assess anxiety severity, symptomatology, and functional impairment. Each item is again scored on a 0- to 3-point basis, and total scores can range from 0 to 21 with greater scores indicating greater severity of anxiety symptoms. As on for the PHQ-9, a total cutoff score of at least 10 has been recommended to detect clinical levels of anxiety symptom severity [22]. Participants again were asked to complete this measure after each CBI session. Internal consistency for GAD-7 scores in this study was acceptable (α=.71 at the first session).

#### Group Session Ratings Scale

The Group Session Ratings Scale (GSRS) is a 4-item self-report measure of the working alliance measured on 10-point sliding scale [24]. Total scores can range from 4 to 40 with higher scores indicating greater levels of the group alliance. It has demonstrated acceptable validity and reliability [24,25]. Participants again were asked to complete this measure after each CBI session. Internal consistency for GSRS scores in this study was good (α=.85 at the first session).

#### Online Social Support Scale

The Online Social Support Scale (OSSS) is a 40-item self-report measure of web-based social support one has received from sessions [26]. We removed 11 items that did not apply to the current intervention, including items that covered instrumental support (eg, the provision of financial aid, material resources, and needed services). Therefore, participants completed a 29-item version of the OSSS, measuring constructs of esteem or emotional support, social companionship, and informational support. Each item is scored from 1 to 5, and total scores on our 29-item version could range from 29 to 145 with higher scores reflecting greater web-based social support. The OSSS has demonstrated evidence of reliability and validity [26]. Participants were asked to complete this measure only once per month. Internal consistency for OSSS scores in this study was excellent (α=.98 at the first session).

### CBI Intervention and Coach Training

CBI was conducted in a metaverse app called Innerworld, which was developed by Innerworld, Inc. [13]. Participants could connect to Innerworld with an Oculus Quest VR head-mounted display, a desktop computer, or mobile phone. Participants are depicted through customizable avatars with anonymous usernames. Participants can view each other’s avatars and body movements while communicating with their actual voices.

The CBI program was created based on the principles of the cognitive behavioral model [4]. Innerworld, Inc handled all recruitment and training of coaches providing CBI. All coaches had to be at least 18 years of age, but no prior professional training in psychology or therapy was required for coaches prior to undergoing CBI training. Coaches received a minimum of 18 hours of intensive training in cognitive behavioral principles, cognitive behavioral techniques, basic psychological skills (eg, using open-ended questions and developing an empathetic tone), and ethics training. Coaches also participated in ongoing weekly supervision. Each CBI session began with a “check-in” during which each participant had the opportunity to share a recent challenge and success followed by a discussion on a cognitive behavioral tool. CBI sessions were offered daily, and participants were encouraged to attend as many sessions as they liked.

### Procedures

No specific recruitment efforts took place to recruit participants for this study. Upon joining Innerworld, participants were provided a tutorial that explained the app’s routine data collection and monitoring for the purposes of quality improvement and research. The tutorial also noted that the app had funding from the National Institutes of Health. Following the tutorial and before being able to participate in CBI, participants were required to provide consent to these data collection efforts and to agree to Innerworld, Inc’s privacy policy and terms of service. In addition to following the tutorial, applicants were asked to complete an optional demographics survey that appeared as a pop-up window within the Innerworld app.

CBI sessions were available daily, and participants were encouraged to attend as many CBI sessions as they liked. Symptom and interpersonal measures were distributed to participants through pop-up windows that appeared in the app interface. See Table 2 detailing the timing of measure distribution. No incentive was provided for the completion of measures. In addition to these self-report measures, the Innerworld app also automatically tracked the number of sessions attended and the type of device participants used to enter the app.

**Table 2:** Timing of measure distribution.

Measure			Time of distribution
Demographics survey			Upon entering Innerworld app
**Symptom measures**			
	PHQ-9^a^	After each CBI^b^ session	
	GAD-7^c^	After each CBI session	
**Interpersonal measures**			
	GSRS^d^	After each CBI session	
	OSSS^e^	Once per month	

^a^PHQ-9: 9-item Patient Health Questionnaire.

^b^CBI: Cognitive Behavioral Immersion.

^c^GAD-7: 7-item Generalized Anxiety Disorder scale.

^d^GSRS: Group Session Ratings Scale.

^e^OSSS: Online Social Support Scale.

### Ethical Considerations

Innerworld, Inc passed only deidentified data to this study’s researchers for the purpose of quality improvement as determined by Vanderbilt University’s institutional review board (#200327).

### Analytic Strategy

#### Changes in Symptoms Across CBI

A repeated measures ANOVA was conducted to examine differences in symptom scores between each session on the intent-to-treat sample. We conducted a model for each of the 2 symptom measures (ie, PHQ-9 and GAD-7). Given that there was a considerable amount of variability in the number of sessions each participant completed and subsequently the number of measures completed, the session comparisons were limited in the repeated measures ANOVA to the first 10 sessions each participant may have completed. We chose this cutoff because most participants (n=90, 70.1%) completed 10 sessions or less. Based on the recommendation of statisticians analyzing the best ways to handle missing values when conducting an intent-to-treat analysis, it was decided to take a mixed model approach without any ad hoc imputation to run this analysis [27]. We report η_p_^2^ as a measure of effect size for these analyses. These models were conducted using PROC MIXED in Statistical Analysis System (SAS; version 9.4; SAS Institute).

#### Relation of Interpersonal Variables With Symptom Change

For our analyses concerning whether the interpersonal variables (ie, GSRS and OSSS) predicted change in symptoms, we first calculated residual change scores for each symptom measure. Residual change scores are calculated by taking the residual from a regression model in which first session symptom scores are entered as predictors of the most recent symptom scores. Given the difference in the number of times participants were asked to complete the GSRS and OSSS (ie, once per session for the GSRS and once per month for the OSSS), average GSRS and OSSS scores were calculated for each participant. We then used general linear models to examine the interpersonal variables as predictors of symptom scores (measured as residual change scores) while controlling for the total number of sessions each participant completed. One model was run for each symptom measure. Models were run using PROC GLM in SAS 9.4.

#### Magnitude of Symptom Change Between VR and Non-VR Users

We conducted 2-group repeated measures ANOVAs to examine the effects of device type used to access CBI (VR vs non-VR device) and time (ie, session) on symptom scores. We ran 1 model for each symptom measure. Similar to our previous ANOVA models, the session comparisons were limited to the first 10 sessions. These models were conducted using PROC MIXED in SAS 9.4.

## Results

### Changes in Symptoms Across CBI

A repeated measures ANOVA determined that depression symptom scores differed significantly across sessions (*F*_9,422_=5.19; *P*<.01; η_p_^2^=0.16). A post hoc pairwise comparison using the Tukey correction showed a significant decrease in PHQ-9 scores between a number of sessions, including a significant difference between participants’ first and tenth session (*P*=.002). Notably, participants endorsed moderate to severe depression symptom severity at their first session (mean 16.44, SD 4.77; range 10-27) [21]. Participants’ reports of depression symptoms were significantly lower at the tenth session (mean 11.62, SD 5.59; range 1-24).

**Figure 2:**
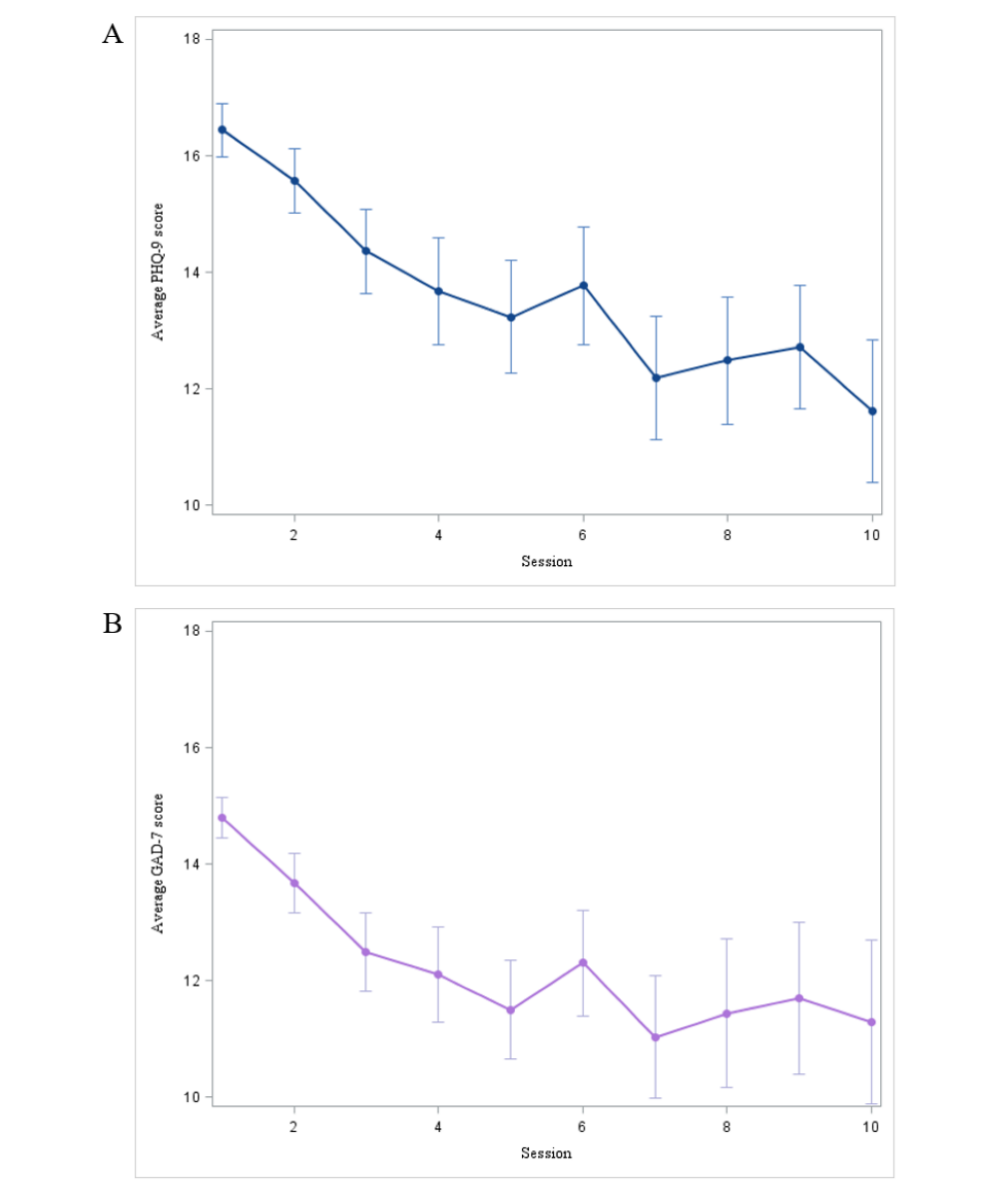
(A) Depression and (B) anxiety symptom change across cognitive behavioral immersion. GAD-7: 7-item Generalized Anxiety Disorder scale; PHQ-9: 9-item Patient Health Questionnaire.

A repeated measures ANOVA also showed that anxiety symptom scores differed significantly across sessions (*F*_9,416_=4.45; *P*<.01; η_p_^2^=0.26). After applying the Tukey correction, we also found a significant decrease in GAD-7 scores between a number of sessions, including a significant difference between participants’ first and tenth session (*P*<.01). Similar to the depression scores, participants also tended to endorse moderate to severe anxiety symptom severity at their first session (mean 14.79, SD 3.45; range 10-21) [22]. Participants’ reports of anxiety symptoms were substantially lower at their 10th session (mean 11.29, SD 6.42; range 0-21). A table of the significant session comparisons from Tukey post hoc comparisons can be found in Multimedia Appendix 1. See Figure 2 for a graph charting average symptom scores across the first 10 sessions.

### Relation of Interpersonal Variables With Symptom Change

We next examined whether alliance or web-based social support (represented by average GSRS and OSSS scores across the course of treatment) predicted change in depression symptoms (represented by a residual change in PHQ-9 scores from participants’ first to most recent session) while controlling for participants’ total number of sessions attended. We found a significant effect for OSSS scores predicting change in PHQ-9 scores, such that greater OSSS scores predicted greater symptom improvement (β_standardized_=–1.50; β=–.07; SE=0.03; t_85_=–2.06; *P*=.04). However, GSRS scores did not significantly predict change in PHQ-9 scores (β_standardized_=–.69; β=–.08; SE=0.08; t_85_=–0.94; *P*=.35).

We next examined whether the alliance or web-based social support (averaged GSRS and OSSS scores across the course of treatment) predicted change in anxiety symptoms (represented by a residual change in GAD-7 scores from participants’ first to most recent session). Neither OSSS (β_standardized_=–.14; β=–.04; SE=0.03; t_79_=–1.11; *P*=.27) nor GSRS scores predicted change in GAD-7 scores (β_standardized_=.04; β=.02; SE=0.09; t_79_=0.28; *P*=.78).

### Magnitude of Symptom Change Between VR and Non-VR Users

We first conducted a 2-group repeated measures ANOVA to examine the effects of VR versus non-VR usage and session on PHQ-9 scores. We did not find a significant main effect of device type used on PHQ-9 scores (*F*_1,105_=2.24; *P*=.14; η_p_^2^=0.002) or a significant interaction between device type used and session (*F*_9,413_=1.79; *P*=.07; η_p_^2^=0.005). We then conducted a 2-group repeated measures ANOVA to examine the effects of VR versus non-VR usage and session on GAD-7 scores. The results of this analysis revealed a significant main effect of device type used on GAD-7 scores (*F*_1,97_=4.14; *P*=.04; η_p_^2^=0.002), indicating that there was a significant difference in anxiety symptoms between participants who used a VR head-mounted display to access CBI versus to those who did not, regardless of time. Further examination of the least squares means revealed that, on average, participants who used VR head-mounted displays had lower anxiety symptoms than those who did not at nearly every session (the exception being session 7). Of note, we did not find a significant interaction between device type used and session (*F*_9,407_=1.54; *P*=.13; η_p_^2^=0.04).

## Discussion

### Principal Findings

Given recent advances in technology, the metaverse has been transformed from a science fiction idea into a reality [28]. Because of its seemingly limitless possibilities, the metaverse is gaining attention across many fields including the mental health field [29]. Using the metaverse has the potential to improve the accessibility and affordability of mental health interventions while maintaining their fidelity and (most importantly) their efficacy. Several metaverse apps are being built to seize the opportunities of this technology, but there remains little regulation as to what they do or knowledge regarding their effectiveness. To our knowledge, this study is the first to evaluate symptom outcomes in a mental health intervention conducted in the metaverse. We found evidence of significant reduction in depressive (η_p_^2^=0.16) and anxiety symptoms (η_p_^2^=0.26) across CBI—a novel coach-led cognitive behavioral skills training group conducted in the metaverse. Given the absence of a control group, we cannot attribute these reductions to CBI, but their magnitude was impressive and, in the range, reported in the literature for bona fide treatments [30,31]. This is especially exciting given that the coaches who were trained to lead CBI sessions were not therapists and required no prior professional training in providing mental health services. We are preparing to conduct a randomized controlled trial to evaluate the efficacy of CBI, but these initial pilot data suggest that such a trial is worthy of pursuit.

The primary aim of this study was to examine symptom change across the course of CBI participation, but we were also interested in the process through which these effects might be achieved. One unique contribution of the metaverse as compared to many previous digital modalities is the ability to interact with others while engaging in the electronic app. In doing so, interpersonal elements that so often play an important role in psychological interventions (ie, nonspecific effects) can be maintained [18]. Therefore, we assessed participant’s perception of the working alliance and web-based social support. We found that only participants’ perceived sense of web-based social support was significantly related to depressive symptom improvement, while neither interpersonal variable predicted anxiety symptom change. Both the alliance and social support are similar in that they are interpersonal variables, but it appears that the relationship that is fostered with peers in the web-based community may play a more important role in symptom improvement as compared to the group alliance, particularly in the context of alleviating depression symptoms.

Another strength of CBI is its compatibility to be accessed with different devices, including VR head-mounted displays that can create a truly immersive experience for participants who enter the metaverse. The vast majority (n=100, 78.7%) of participants in the sample for this study used a VR head-mounted display to participate in CBI, while the remainder accessed CBI through the use of their smartphone or desktop computer. Although we did not randomly assign the device participants used, we found that participants who tended to use the VR head-mounted displays also reported a greater magnitude of anxiety symptom change on average than those who used non-VR devices at nearly every session. It may be possible that the level of immersion or extent to which a participant interacts with their virtual environment as if it was real [14] may be an important element of digital interventions for these symptoms. Future research is needed to further explore the effect that the extent of immersion may have on CBI outcomes and other metaverse mental health interventions. Outcomes from such research could be used to assess whether the increased cost of a head-mounted display may be warranted to lead to better outcomes.

Notably, our sample was predominantly male (n=68, 53.5%). This contrasts with the predominantly female composition of typical treatment-seeking samples [32,33]. Men are typically less likely to seek treatment than women, thus their mental health is often overlooked and undertreated. This is a critical problem, especially due to the troubling fact that depression and suicide are among the leading causes of death among men [34]. Thus, our sample composition is especially exciting because it suggests that the metaverse may be a particularly accessible and appealing setting for men to access mental health services, potentially increasing the reach of mental health intervention for this underserved population.

### Limitations

We note some key limitations of this study. First, we used self-report measures of the alliance, web-based social support, and symptoms. While each of the self-report measures used in this study has been validated, there are alternative measurement approaches that can provide more nuanced assessments of these variables. For example, researchers suggest that the congruence between multiple perspectives in rating the alliance may provide more useful information than a single perspective alone [35,36] and that observer-rated alliance may provide an even less biased perspective that is more related to outcomes [37]. Relatedly, we only assessed 2 interpersonal variables and 2 symptom measures. Given the transdiagnostic nature of the CBI program, additional process and outcome variables will be important to assess. We also believe it will be important to assess attitudes related to the provision of mental health services in the metaverse, as attitudes can affect interest in, engagement with, and expectations of the intervention. Second, we cannot establish causality of relations between variables in this study. Without a control group, we are unable to say with certainty that participation in CBI causes symptom change. Given the timing of assessments, we are unable to say whether the alliance or web-based social support occurred prior to the symptom change with which it was associated. It will be important for future studies to consider the timing of assessments. It will also be useful to consider confounding variables that might influence these process-outcome relations (such as educational levels of coaches and participant demographic variables). Third, our sample was comprised of predominantly White adults 50 years or younger of age. Therefore, there should be caution in generalizing these findings to other samples with respect to ethnicity or race and age (17 years or younger and 50 years or older).

### Future Directions

Given the significant depression and anxiety symptom improvements our sample experienced, it will be important to continue examining the process of change so we can continue to promote and improve these outcomes. Nonspecific effects are one avenue through which symptom outcomes can be achieved [18]. However, specific factors such as cognitive or behavioral techniques are also frequently examined process variables that can elevate symptom change [38]. Although our research group is aware of the methodological challenges of rating process variables through rigorous traditional observer-coding projects [39], the digital nature of this intervention may open another future opportunity to automate and enhance the coding procedures of process variables. Future studies are encouraged to not only measure additional process variables but also to pay special attention to the timing of variables to ensure temporal precedence of variables in line with causal relations. This will allow us to better understand the process of change as well as elucidate mediators and moderators of process-outcome relations [40].

Moreover, it will be important for future researchers to investigate metaverse mental health interventions in more diverse samples. The CBI intervention can be accessed virtually anywhere at any time, so it has the potential to reach various populations who may otherwise not have access to empirically supported mental health services. More targeted recruitment of underserved populations is needed.

One exciting feature about the Innerworld app used in this study is that it was designed to be a clinical research platform that welcomes research collaborations to continue investigating these relations with sophisticated methods and continuous data collection. We encourage collaborative efforts with team expertise in both technology and therapeutic processes to continue the investigation into and enhancement of metaverse mental health interventions.

### Conclusions

This study evaluated the symptom outcomes in a sample of individuals who participated in a coach-led cognitive behavioral skills training group conducted in the metaverse. These participants experienced significant depression and anxiety symptom improvements, and these improvements were related to higher levels of web-based social support. With the increasing popularity of the metaverse and VR, we strongly encourage researchers to continue investigating its potential for further accessibility, affordability, and efficacy of mental health interventions.

## Appendix

Multimedia Appendix 1 Results from Tukey post hoc comparisons.

## Abbreviations

CBI: Cognitive behavioral immersion
CBT: cognitive behavioral therapy
GAD-7: 7-item Generalized Anxiety Disorder scale
GSRS: Group Session Ratings Scale
OSSS: Online Social Support Scale
PHQ-9: 9-item Patient Health Questionnaire
SAS: Statistical Analysis System
VR: virtual reality
