# Supplementary material for: Examining Predictors of Depression and Anxiety Symptom Change in Cognitive Behavioral Immersion: Observational Study
Source: JMIR Ment Health. 2023 Jul 14;10:e42377. doi: 10.2196/42377 (PMC10382949; doi:10.2196/42377)
Supplement: Multimedia Appendix 1 [file mental_v10i1e42377_app1.docx]

**Multimedia Appendix 1: Results from Tukey post hoc comparisons**

Significant depression symptom differences between sessions

| Differences of Least Squares Means | | | | | |
| --- | --- | --- | --- | --- | --- |
| Session comparison | Estimate | SE | DF | t Value | Tukey P |
| 1, 3 | 2.16 | 0.59 | 422 | 3.65 | .01 |
| 1, 4 | 2.83 | 0.64 | 422 | 4.43 | <.001 |
| 1, 5 | 2.95 | 0.69 | 422 | 4.27 | .001 |
| 1, 7 | 3.49 | 0.80 | 422 | 4.37 | <.001 |
| 1, 8 | 3.41 | 0.83 | 422 | 4.11 | .002 |
| 1, 9 | 3.19 | 0.88 | 422 | 3.62 | .01 |
| 1, 10 | 3.95 | 0.95 | 422 | 4.18 | .002 |
| 2, 7 | 2.62 | 0.80 | 422 | 3.28 | .04 |
| 2, 10 | 3.08 | 0.95 | 422 | 3.26 | .04 |

Significant anxiety symptom differences between sessions

| Differences of Least Squares Means | | | | | |
| --- | --- | --- | --- | --- | --- |
| Session comparison | Estimate | SE | DF | t Value | Tukey P |
| 1, 3 | 2.26 | 0.63 | 416 | 3.59 | .01 |
| 1, 4 | 2.74 | 0.67 | 416 | 4.08 | .002 |
| 1, 5 | 3.27 | 0.72 | 416 | 4.51 | <.001 |
| 1, 7 | 3.49 | 0.83 | 416 | 4.19 | .001 |
| 1, 8 | 3.25 | 0.86 | 416 | 3.77 | .01 |
| 1, 9 | 2.94 | 0.91 | 416 | 3.22 | .04 |
| 1, 10 | 3.50 | 1.00 | 416 | 3.51 | .02 |
